# Health promotion in individuals with mental disorders: a cluster preference randomized controlled trial

**DOI:** 10.1186/1471-2458-13-657

**Published:** 2013-07-15

**Authors:** Nick Verhaeghe, Els Clays, Carine Vereecken, Jan De Maeseneer, Lea Maes, Cornelis Van Heeringen, Dirk De Bacquer, Lieven Annemans

**Affiliations:** 1 Department of Public Health, Ghent University, Ghent, Belgium; 2 Department of Family Medicine and Primary Health Care, Ghent University, Ghent, Belgium; 3 Department of Psychiatry and Medical Psychology, Ghent University, Ghent, Belgium; 4 Faculty of Medicine and Pharmacy, Vrije Universiteit Brussel, Brussels, Belgium

**Keywords:** Mental health care, Health promotion, Physical activity, Healthy eating, Intervention

## Abstract

**Background:**

The existing literature on weight management interventions targeting physical activity and healthy eating in mental health care appears to provide only limited evidence. The aim of the study was to examine the effectiveness of a 10-week health promotion intervention, followed by a 6-month follow-up period in individuals with mental disorders living in sheltered housing in the Flanders region (Belgium).

**Methods:**

The study had a cluster preference randomized controlled design. Twenty-five sheltered housing organisations agreed to participate (16 in the intervention group, nine in the control group). In the intervention group, 225 individuals agreed to participate, while in the control group 99 individuals entered into the study. The main outcomes were changes in body weight, Body Mass Index, waist circumference and fat mass. Secondary outcomes consisted of changes in physical activity levels, eating habits, health-related quality of life and psychiatric symptom severity.

**Results:**

A significant difference was found between the intervention group and the control group regarding body weight (−0.35 vs. +0.22 kg; p=0.04), Body Mass Index (−0.12 vs. +0.08 kg/m^2^; p=0.04), waist circumference (−0.29 vs. + 0.55 cm; p<0.01), and fat mass (−0.99 vs. −0.12%; p<0.01). The decrease in these outcomes in the intervention group disappeared during the follow up period, except for fat mass. Within the intervention group, a larger decrease in the primary outcomes was found in the participants who completed the intervention. No significant differences between the two groups in changes in the secondary outcomes were found, except for the pedometer-determined steps/day. In the intervention group, the mean number of daily steps increased, while it decreased in the control group.

**Conclusions:**

The study demonstrated that small significant improvements in the primary outcomes are possible in individuals with mental disorders. Integration of health promotion activities targeting physical activity and healthy eating into daily care are, however, necessary to maintain the promising results.

**Trial registration:**

This study is registered at ClinicalTrials.gov NCT 01336946

## Background

Several studies have shown that individuals with mental disorders (MD) including schizophrenia, bipolar disorders, depression, and anxiety disorders are at a greater risk of being overweight (Body Mass Index 25–29.9 kg/m^2^) or obese (Body Mass Index ≥30 kg/m^2^) than the general population [1-3]. These conditions and other related metabolic disturbances [4,5] are substantial risk factors for the high prevalence of cardiovascular disease (CVD) and associated mortality in this population [6,7].

In people with MD, excessive weight gain has been associated with the use of especially second generation antipsychotics (SGAs), the degree of which is variable according to the type of antipsychotic drug used [8,9]. Beside the side effects of these drugs, the high prevalence of overweight and obesity in this population is also associated with lower physical activity (PA) levels and more unhealthy eating habits. The former include less moderate or vigorous PA than the general population or PA guidelines [10-12], the latter include eating fresh fruit, vegetables, wholegrain bread and, milk products less frequently, eating more instant meals [13-15] and fat [16], and having less variety of food in the diet [17]. Growing attention is being given to lifestyle interventions targeting PA and healthy eating, the effectiveness of which in the general population is already well established [18,19]. The current literature on weight reduction interventions in mental health care appears to provide only limited evidence of the effectiveness of either psycho-educational programmes or programmes including educational and exercise components [20]. More research is therefore required to examine the effectiveness of these kinds of interventions in populations of individuals with MD.

This paper describes the results of a health promotion intervention targeting PA and healthy eating in individuals with MD living in sheltered housing in the Flanders region (Belgium). The study consisted of a 10-week intervention period followed by a 6-month follow up period.

## Methods

### Study design and setting

The study design consisted of a cluster preference randomized controlled trial (RCT). The study was conducted in sheltered housing organisations (SHOs) in the Flanders region (Belgium), where there were 42 SHOs amounting to 2662 approved places at the time [21].

In Belgium, sheltered housing is aimed at people with MD aged 18 years and older who do not need to stay in a psychiatric inpatient facility on a permanent basis. The main goal is the psychiatric rehabilitation of the patients (usually known as ‘residents’). In the rehabilitation process, patients receive support to develop the emotional, social and intellectual skills needed to live, learn and work in the community with the lowest amount of support [22]. In practice, the usual treatment consists of weekly meetings between the mental health nurse and the resident to discuss topics such as how to cope with the psychiatric disease, somatic health, household tasks, and financial issues. The main objective is to maximize the personal autonomy of each resident who is encouraged to do as much as possible for him/herself including self-care, shopping, cooking, domestic chores and leisure time activities with the support of the mental health nurses. Particular attention is given to the search for a meaningful daily occupation such as regular or sheltered employment, or voluntary work.

Residents can live alone or they can live together with other patients in ‘community houses’. In these settings, residents have their own bedrooms but share communal areas such as the kitchen, bathroom and living room. Both individuals living alone as well as those living in ‘community houses’ were eligible for participation. Assignment to the intervention or control group at the individual level was not possible because of the high risk of contamination bias by participants living together in the same ‘community house’. This risk of bias was avoided as the assignment to the intervention or control group occurred at the level of the SHO. Based on the literature [23,24] on possible problems in the design of studies evaluating behavioural or psychosocial interventions, it was assumed that being assigned to a non-preferred intervention condition could reduce the SHOs interest in participating in the study. For this reason, it was decided to use a preference design. In this design, subjects are allowed to select the intervention assignment. This is useful when strong preferences among potential participants threaten either the ability to recruit an adequate sample size of representative participants or when such preferences threaten participants’ acceptance of treatment assignment, adherence or retention in the trial [25]. In this study, SHOs with strong preferences were offered their preferred condition, while those without were randomized in the usual way.

### Study population and recruitment

The study population consisted of individuals with MD aged between 18 and 75 years of age living in sheltered housing in the Flanders region. Exclusion criteria included individuals older than 75 years, those fitted with a gastric ring or pacemaker and those with cognitive impairments which would prevent them being able to follow and understand the psycho-educational and behavioural sessions of the health promotion intervention (as assessed by the mental health nurses).

Recruitment of participants took place from June 2010 until January 2011. An invitation letter and response form with a self-addressed stamped envelope was sent to the administrators of all SHOs in the Flanders region. The letter included a concise explanation of the aim of the study and the expectations and content if an organisation was willing to serve as intervention group. The administrators were asked to discuss the study proposal with their staff to decide whether they were interested in participating. Involving the staff members in the decision process was considered important because, if an organisation was willing to serve as intervention group, one or more mental health nurses would be involved in the intervention. They were asked if they were interested in participating in the study with (i) no preference to serve as intervention or control group and to be randomized or, (ii) a preference to serve as intervention group or, (iii) a preference to serve as control group. SHOs which did not respond after six weeks were sent a reminder. SHOs which were not prepared to participate were asked to report the reason for non-participation. The administrators and staff were also instructed to not consult the residents in the decision process.

Based on responses to the letter, the SHOs were subsequently either assigned to the intervention group or control group according to their preference or, when no preference had been expressed, randomly assigned to the intervention or control group. Randomization occurred by means of simple randomization using a lottery method conducted by an external person not involved in the study.

Finally, the residents living in the intervention and control SHOs received both written and oral information about the study. The written information consisted of a detailed explanation of the study and an informed consent. The content of these documents was also orally explained to the residents by the mental health nurses.

### Development of the materials

The theoretical framework for the intervention is based on elements of social-cognitive theory [26], self-determination theory [27], and control theory [28]. The intervention was developed using the mediating variable approach including the mediating variables of knowledge, attitude, skills, self-efficacy and motivation [26,27]. For instance, based on the social-cognitive theory, it was decided to develop a workshop with sessions spread over 10 weeks as this stimulates interaction and exchange of experiences among the participants and with the workshop leader. Based on the self-determination theory and control theory, an emphasis was put on the motivation to change lifestyle through discussions on the advantages and disadvantages of healthy eating and PA on quality of life and long term health benefits. Individual support was also available and participants were stimulated to define specific personal goals for eating habits (for example: eating less energy dense food) or engaging in PA. These personal goals could be further discussed and followed up with the nurses.

A detailed staff manual was developed based on the manual ‘Health promotion on well-balanced eating and healthy physical activity’ developed by the Flemish Institute of Health Promotion and Disease Prevention [29]. The target population of this manual was the general population. For this reason, some adjustments were made to the manual to better meet the needs of our study population (e.g. how to live a healthier lifestyle despite the presence of barriers associated with the MD such as the sedative effects of certain psychotropic drugs). The manual was built around ten themes regarding PA and healthy eating: 1) PA and healthy eating: Introduction; 2) Awareness of the consumption of fat and fibres; 3) A healthy lifestyle: advantages and barriers; 4) The Active Food Triangle; 5) Using the Active Food Triangle throughout the day; 6) Label reading; 7) The influence of the environment & Budget issues; 8) & 9) Physical activity; 10) A quiz on PA and healthy eating.

### Implementation of the intervention

All sheltered housing organisations in the intervention group were visited by one member of the research team (NV). The aim of the visit was to instruct the mental health nurses who would be offering and supervising the group-based sessions. During a 2-hour training session they received background information about the burden of obesity in individuals with MD and the importance of health promotion targeting PA and healthy eating, and were instructed how to use the staff manual and how to organise the walking sessions. Other staff members were also asked to attend this training session because information and instructions concerning the “individual support” was also provided. The nurses were instructed to provide individual support to all the participants during the 10-week health promotion intervention. To minimize the workload for the nurses, they were asked to provide this individual support during their weekly meeting with the resident. During the 10-week intervention, the nurses could contact one of the researchers by phone or by e-mail if necessary.

### Study duration and intervention components

The study period consisted of an intervention period of 10 weeks followed by a post-intervention period of 24 weeks. In addition to treatment as usual, the intervention group received the 10-week health promotion programme, while the control group received only treatment as usual. The programme was delivered by one or more mental health nurses working in the intervention SHOs. All participants received the same information in the same format comprising the following components:

•Psycho-educational and behavioural group sessions

•A weekly group session including discussions on PA and healthy eating, problem solving, written exercises, quizzes and plans to increase PA levels and to stimulate healthier eating behaviour.

•Supervised exercise

•In the same 10-week period a weekly 30-minutes supervised walking session was organised.

•Individual support

•During the 10-week intervention period, the participants in the intervention group received individual support from the mental health nurses. These sessions lasted about ten minutes and the following issues were discussed: (i) “Did you understand what was discussed during the group-based sessions?”, (ii) “How difficult was it for you to follow the advice given during the sessions?”, and (iii) “What made it difficult for you to follow the advice and what can be done about it?”. The nurses were also asked to inform the participants about the practical issues concerning the next session such as date, time and location.

### Sample size calculation

The sample size calculation was based on an average change of the primary outcome body weight of 3.5 kg between the intervention and control group at the end of the study. This change was based on the results of a literature review performed by the research team [30]. Cluster randomized trials require larger sample sizes than the individually randomized design because observations on individuals in the same cluster tend to be correlated, and so the effective sample size is less than the total number of individual participants. This reduction in effective sample size and the degree of correlation within clusters is expressed as the intraclass correlation coefficient (ICC) [31]. The ICC can be interpreted as the proportion of group-level variance compared to the total variance. As no ICC for this kind of intervention in people with MD was found in the literature, an assumption was made by multiplying the sample size by a design factor of 1.5 [32]. A sample size of 371 individuals in each group provided a sample size large enough to detect a difference in mean body weight change of 3.5 kg across the two groups with 80% power at a significance level of 0.05.

### Data collection and outcome measurements

#### Sociodemographics

Participants were asked to complete a sociodemographic questionnaire on sex, age, duration of stay in sheltered housing, marital status, occupational status, contacts with relatives, tobacco and alcohol use, and medication use.

#### Primary outcome measures

The primary outcomes of the study consisted of changes in body weight, Body Mass Index (BMI), waist circumference (WC) and fat mass. Body weight and fat mass were measured in all participants wearing light clothing without shoes using a TANITA BC-420 SMA digital weighing scale (TANITA, Tokyo, Japan). The measurement had to be taken with bare feet as the equipment sends a weak electrical current through the body to measure impedance (electrical resistance) of the body. Fat within the body allows almost no electricity to pass through. The degree of difficulty with which electricity passes through a substance is known as the electrical resistance, and the percentage of fat can be inferred from measurements of this resistance. To prevent a possible discrepancy in measured values, participants had to keep still during measurement. Height was measured in a standardized way using a Seca 225 stadiometer (Seca GmbH & KG, Hamburg, Germany). BMI was calculated by dividing the body weight in kilograms by the square of the height in meters. WC was measured with a Seca 200 tape (Seca GmbH & KG, Hamburg, Germany) according to the ‘Clinical guidelines on the identification, evaluation, and treatment of overweight and obesity in adults’ [33]. All measurement procedures were conducted by a member of the research team. Both the stadiometer and the weighing scale were placed on a flat surface to assure correct measurement of the outcomes.

#### Secondary outcome measures

Changes in PA were assessed using the Dutch version of the self-administered International Physical Activity Questionnaire (IPAQ), which has been shown to be a reliable and valid PA measurement tool [34]. PA levels were also assessed with pedometers using the Yamax Digiwalker SW-200 (Yamax, Tokyo, Japan), as this is known to be accurate and reliable for counting steps [35]. Participants were asked to fill out the number of daily steps during seven consecutive days on a pre-printed document. If necessary they were assisted by a mental health nurse. The dietary habits of the participants were assessed using an adapted version for adults of an online dietary assessment tool, the ‘Young Children’s Nutrition Assessment on the Web’ [36]. Dietary data were collected for two non-consecutive days, one week day and one weekend day. In this tool, each day is distributed in 24 possible eating occasions. The participants were asked to report each food and beverage intake to the nearest hour of the day. For each eating and beverage occasion, the participant selected the foods consumed from a hierarchically organised menu structure, with both the interviewer and the participant sitting in front of the computer screen. Pictures [37] and measurement units (e.g. a spoon, a small bottle) were used to assess portions and portion sizes. For the present analysis all food was grouped into 13 food groups. Health-related quality of life (HRQOL) was examined using the SF-36 Health Survey Questionnaire [38]. Psychiatric symptom severity was assessed using the Brief Symptom Inventory (BSI) [39]. This questionnaire is considered a reliable and valid tool, useful in patient groups with different psychiatric diagnoses [40].

Data on all primary and secondary outcomes were collected at baseline and at ten weeks. At the end of the study (at 36 weeks) data on body weight, WC and fat mass were collected and BMI was calculated. At that time, participants were also asked to complete the SF-36 questionnaire again.

### Data analysis

Depending on the distribution of the quantitative variables, the independent samples T-test or the Mann Whitney U Test were used to compare the intervention and control group at baseline. The X^2^-test was used in qualitative variables.

First, change scores for the primary outcomes of body weight, BMI, WC, and fat mass from baseline to the end of the intervention period (at ten weeks) and from baseline to the end of the study (at 36 weeks) were computed. Next, an intention to treat (ITT) analysis using the independent samples T-test was performed to evaluate differences in the change scores between intervention and control groups in the primary outcome variables from baseline to 10 weeks and from baseline to 36 weeks. ITT analysis included all participants with baseline data. Missing data at 10 and 36 weeks were imputed with the mean change in the primary outcomes in the control group [41]. Univariate analyses of covariance were used to control for sex, age, living situation, smoking habits, alcohol use, psychotropic medication use, and duration of stay in sheltered housing. Additional linear mixed model analysis was performed to take possible clustering effects into account because of the fact that individuals were nested within SHOs. For all primary outcomes, an ICC below 5% was found indicating a limited group-level variance compared to the total variance [42]. In addition, in all participating SHOs the number of residents that agreed to participate was limited to 23 or fewer individuals per SHO. This is less than the ‘30/30 rule’ as suggested by Hox [42]. It is suggested that researchers should strive for a sample of at least 30 groups with a least 30 individuals per group. For these reasons, it was decided to omit mixed model analysis.

To examine whether changes in the primary outcomes were different between participants in the intervention group who completed the health promotion programme (i.e. individuals who attended at least 8 of 10 sessions), those who did not and the controls, a per protocol analysis was performed within the subsample of participants for whom baseline data and data at 10 weeks were available. A Tukey post hoc comparison test was performed to assess whether significant treatment differences between the three groups occurred.

Analyses of the secondary outcomes were based on computed change scores from baseline to the end of the intervention (at ten weeks). Secondary outcome variables were evaluated per protocol using the independent samples T-test or the Mann Whitney U Test depending on the distribution of the quantitative variables to examine differences between the intervention and control groups in changes in these outcomes. A P-value ≤ .05 was considered statistically significant. For statistical analysis, the SPSS®19 statistical software package was used.

### Ethics

Permission to perform the study was obtained from the Ethics Committee of the University Hospital of Ghent (Belgium). Written consent for participation was obtained from all participants. If necessary, the mental health nurse explained the contents of the informed consent document to the candidate, but all candidates had the capacity to consent. Participation in the study was voluntary and all participants were informed that the data analysis would be anonymous and that they could withdraw from the study at any time.

## Results

### Recruitment process and participation rate

**Figure 1:**
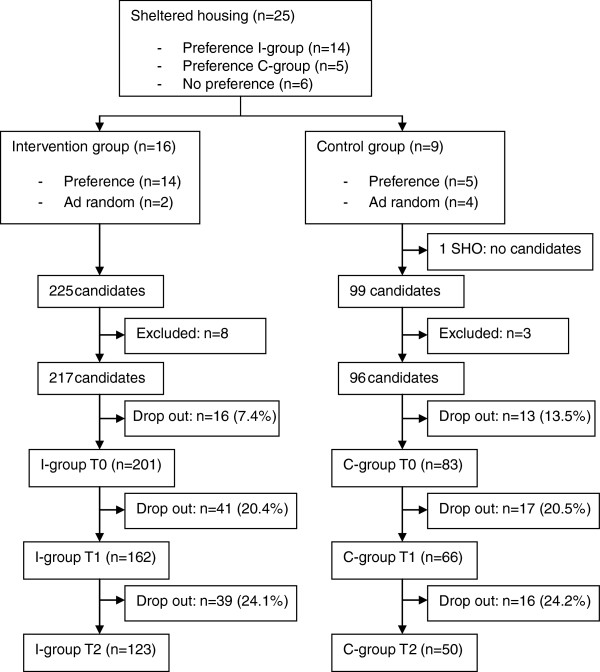
Health promotion intervention: Recruitment process.

In Figure 1 an overview is provided of the recruitment process and participation rate. Twenty-five SHOs agreed to participate, accounting for 59.5% of the total number of SHOs in the Flanders region. Fourteen of these expressed a preference to serve as intervention group, while five preferred to serve as control group. Six had no preference and were randomly assigned to the intervention group (n=2) or to the control group (n=4). In one SHO serving as control group, no residents were interested in participating. On the individual level, 324 residents were willing to engage in the study, 225 and 99 candidates in the intervention and control SHOs respectively. Based on the number of approved places in the participating SHOs, a response rate of 24% in the intervention group and 21% in the control group was obtained.

Eleven candidates were excluded because of age (n=2), cognitive impairments (n=4), having a gastric ring (n=1), or the impossibility of being weighed using the digital weighing scale (n=4). Of these last, three had a pacemaker, and one had an artificial limb. Twenty-nine (9.3%) of the remaining 313 candidates withdrew from the study prior to the baseline measurement. Prior to the baseline measurement a higher proportion of individuals in the control group withdrew from the study (13.5% vs. 7.4%). Dropout rates at ten and 36 weeks were similar in the two groups. From baseline to the end of the study, the most common reasons for withdrawal were no further interest or motivation (n=60), admission to a psychiatric inpatient facility (n=9), and discharge from sheltered housing (n=8). Two-hundred and one individuals in the intervention group and 83 in the control group were included in the ITT analysis.

### Baseline assessment

At baseline, the only significant differences observed between the intervention and control group for the sociodemographic variables were for ‘living situation’, and ‘duration of stay in sheltered housing’ (Table 1). A higher proportion of controls was living together than those in the intervention group (81.0% vs. 62.0%, p=0.002). Median duration of stay in sheltered housing was significantly longer in participants in the intervention group than in the control group (4.4 vs. 2.5 years, p=0.04). Psychotropic medication use was found to be significantly different between the two groups for only the SGAs (p=0.03). Fifty-eight point six per cent and 66.7% of the participants in the intervention and control group respectively were daily smokers. Sixty-eight point two per cent of the participants in the intervention group were on SGA prescription compared to 54.2% in the control group. At baseline, a mean BMI of 30.2 kg/m^2^ and 29.5 kg/m^2^ was observed in the intervention group and control group respectively. No significant differences existed at baseline between the intervention and control group regarding the primary outcome measures (Table 2), nor were any found regarding the secondary outcome measures, except for the food groups soft drinks (p= 0.04) and meat, fish and eggs (p= 0.04) (Tables 3 and 4).

**Table 1:** Baseline sociodemographic data

**Variable**	**Intervention group (n=201)**	**Control group (n=83)**	**p**
Sex, *n (%)*			0.27§
men	119 (59.2)	55 (66.3)	
women	82 (40.8)	28 (33.7)	
Age (years), *mean*±*SD*	46.2±12.5	46.6±11.9	0.83‡
Smoking, *n (%)*			
no smoking	77 (41.4)	26 (33.3)	0.22§
smoking	109 (58.6)	52 (66.7)	
Alcohol use, *n (%)*			
regular	87 (46.5)	35 (44.9)	0.81§
never	100 (53.5)	43 (55.1)	
Employment, *n (%)*			0.51§
regular	11 (5.9)	2 (2.5)	
sheltered	73 (39.2)	32 (40.5)	
no employment	102 (54.8)	45 (57.0)	
Living situation, *n (%)*			<0.01§
alone	71 (38.0)	15 (19.0)	
with others	116 (62.0)	64 (81.0)	
Contact with family, *n (%)*			0.70§
regular	134 (72.0)	58 (74.4)	
seldom/never	52 (28.0)	20 (25.6)	
Contact with friends, *n (%)*			0.12§
regular	111 (60.0)	54 (70.1)	
seldom/never	74 (40.0)	23 (29.9)	
Stay in SH (years), *median (range)*	4.4 (0.1-22.3)	2.5 (0.1-16.3)	0.04†
DSM-IV diagnosis, *n (%)*			
schizophrenia	80 (41.2)	25 (30.1)	0.08§
mood disorder	44 (22.7)	24 (28.9)	0.27§
substance misuse	30 (15.5)	14 (16.9)	0.77§
personality disorder	29 (14.9)	11 (13.3)	0.71§
other	11 (5.7)	9 (10.8)	0.13§
Medication, *n (%)*			
sedatives/anxiolytics	60 (29.9)	34 (41.0)	0.07§
first generation antipsychotics	46 (22.9)	13 (15.7)	0.17§
second generation antipsychotics	137 (68.2)	45 (54.2)	0.03§
antidepressants	106 (52.7)	46 (55.4)	0.68§

§Pearson Chi-Square; ‡ Independent samples t-test; †Mann–Whitney U test; *SH* Sheltered Housing.

*DSM-IV* Diagnostic and Statistical Manual of Mental Disorders.

**Table 2:** Baseline assessment and changes in primary outcomes from baseline to 10 and 36 weeks (ITT)

**Variable**	**Intervention group (n=201)**	**Control group (n=83)**	**Between group change**	**p**^**‡**^
Weight (kg), *mean*±*SD*				
baseline	87.95±20.74	85.19±16.04		0.23
at 10 weeks	87.60±20.77	85.41±16.49		
change at 10 weeks	−0.35±2.15	0.22±2.14	0.57	0.04
≥ 5% weight loss, *n (%)*	11 (5.5%)	3 (3.6%)		
at 36 weeks	88.28±21.03	86.18±16.99		
change at 36 weeks	0.33±3.51	0.99±3.01	0.66	0.14
BMI (kg/m^2^), *mean±SD*				
baseline	30.22±6.14	29.52±5.41		0.37
at 10 weeks	30.10±6.18	29.60±5.56		
change at 10 weeks	−0.12±0.75	0.08±0.78	0.20	0.04
at 36 weeks	30.33±6.26	29.87±5.74		
change at 36 weeks	0.11±1.23	0.35±1.05	0.24	0.14
Waist (cm), *mean*±*SD*				
baseline	106.16±16.81	105.21±14.39		0.65
at 10 weeks	105.87±16.73	105.76±14.72		
change at 10 weeks	−0.29±2.18	0.55±2.61	0.84	<0.01
at 36 weeks	106.32±16.82	106.43±15.10		
change at 36 weeks	0.16±3.41	1.22±3.53	1.06	0.02
Fat mass (%), *mean*±*SD*				
baseline	34.17±10.55	33.37±10.63		0.57
at 10 weeks	33.18±10.62	33.25±10.72		
change at 10 weeks	−0.99±2.51	−0.12±1.60	0.87	<0.01
at 36 weeks	33.76±11.21	34.56±11.04		
change at 36 weeks	−0.41±3.57	1.19±2.58	1.60	<0.001

‡ Independent samples t-test.

ITT, intention to treat; kg, kilogramme; BMI, Body Mass Index; cm, centimetre.

**Table 3:** Baseline assessment and changes in secondary outcomes from baseline to 10 weeks

**Variable**	**Intervention group**	**Control group**	**Between group change**	**p**
Total PA^1^ (METmin/week), *mean*±*SD*				
baseline	3282±3118	3770±3071		0.20†
at 10 weeks	4992±5089	4497±4586		
change	1709±4430	727±4887	982	0.17‡
Moderate PA^1^ (METmin/week), *mean±SD*				
baseline	1568±1990	2061±2334		0.07†
at 10 weeks	2518±3026	2346±3479		
change	950±2849	285±3842	665	0.19‡
Vigorous PA^1^ (METmin/week), *mean*±*SD*				
baseline	486±2036	311±853		0.74†
at 10 weeks	979±2992	591±1559		
change	493±2445	279±1619	214	0.54‡
Walking PA^1^ (METmin/week), *mean*±*SD*				
baseline	1228±1441	1398±1564		0.44†
at 10 weeks	1495±1617	1560±1687		
change	266±1988	163±2096	103	0.74‡
Pedometer^2^ determined steps/day, *mean±SD*				
baseline	6872±3585	7215±3368		0.61‡
at 10 weeks	8128±4050	6788±3507		
change	1256±1933	−426±2754	1340	<0.001‡
SF36 PCS^3^, *mean*±*SD*				
baseline	40.4±7.7	41.2±5.9		0.51‡
at 10 weeks	39.4±7.5	40.2±7.8		
change	−0.9±8.5	−1.0±7.6	0.1	0.96‡
SF36 MCS^3^, mean±SD				
baseline	35.6±8.7	35.8±7.9		0.89‡
at 10 weeks	34.8±7.9	35.3±7.4		
change	−0.9±7.6	−0.5±6.8	0.4	0.77‡
BSI_PST^4^, *mean*±*SD*				
baseline	27.6±12.6	26.5±13.8		0.60‡
at 10 weeks	25.3±12.8	24.0±14.5		
change	−2.4±9.5	−2.5±7.5	0.1	0.92‡

PA, physical activity; PCS, physical component score; MCS, mental component score; BSI_PST, Brief Symptom Inventory Positive Symptom Total.

^1^Intervention group, n=127; Control group, n=60; ^2^ Intervention group, n=92; control group, n=40; ^3^Intervention group, n=112; control group, n=51; ^4^Intervention group, n=116; control group, n=55.

‡Independent Samples T-test, †Mann Whitney U test.

**Table 4:** Baseline assessment and changes (mean - range) in food intake (g/day) from baseline to 10 weeks

**Variable**		**Intervention group (n=152)**	**Control group (n=49)**	**Between group change**	**p**
potatoes	baseline	96.0 (0–472.5)	99.8 (0–332.5)		0.59†
	at 10 weeks	95.3 (0–250.0)	82.6 (0–195.0)		
	change	−0.64	−17.24	16.6	0.28‡
alcohol	baseline	38.9 (0–1240.0)	38.3 (0–625.0)		0.64†
	at 10 weeks	31.6 (0–1155.0)	56.0 (0–915.0)		
	change	−7.27	17.70	24.97	0.34‡
bread & rolls	baseline	133.0 (0–702.0)	141.2 (0–420.0)		0.32†
	at 10 weeks	124.3 (0–360.0)	119.3 (0–283.5)		
	change	−8.73	−21.97	13.24	0.38‡
soft drinks	baseline	338.9 (0–2900.0)	428.1 (0–2665.0)		0.04†
	at 10 weeks	288.1 (0–2150.0)	492.2 (0–2700.0)		
	change	−50.82	64.16	114.98	0.14‡
fruit	baseline	97.2 (0–1247.5)	84.1 (0–625.0)		0.65†
	at 10 weeks	112.6 (0–1040.0)	73.9 (0–352.5)		
	change	15.42	−10.14	25.56	0.24‡
vegetables	baseline	94.3 (0–690.0)	111.6 (0–1127.5)		0.96†
	at 10 weeks	85.5 (0–520.0)	68.5 (0–430.0)		
	change	−8.87	−43.05	34.18	0.23‡
coffee & tea	baseline	696.4 (0–4125.0)	761.3 (0–2925.0)		0.73†
	at 10 weeks	542.9 (0–2475.0)	632.9 (0–2625.0)		
	change	−153.49	−128.36	25.13	0.78‡
milk	baseline	152.6 (0–1087.5)	212.6 (0–1500.0)		0.48†
	at 10 weeks	143.4 (0–1437.5)	159.4 (0–650.0)		
	change	319.20	−53.17	372.37	0.52‡
sweets	baseline	52.4 (0–385.5)	69.5 (0–552.0)		0.05†
	at 10 weeks	28.4 (0–169.0)	45.5 (0–277.0)		
	change	−24.0	−24.0	0	0.99
water	baseline	515.4 (0–2541.3)	480.3 (0–2860.0)		0.33†
	at 10 weeks	478.3 (0–2212.5)	595.9 (0–2800.0)		
	change	−37.11	115.61	152.72	0.10‡
meat, fish, eggs	baseline	130.0 (0–658.0)	150.5 (0–359.0)		0.04†
	at 10 weeks	148.7 (0–461.0)	139.3 (0–402.0)		
	change	18.19	−11.20	29.39	0.09‡
fatt & sauces	baseline	53.8 (0–287.5)	54.6 (0–347.5)		0.81†
	at 10 weeks	39.3 (0–215.0)	42.2 (0–140.0)		
	change	−14.57	−12.67	1.9	0.85‡
biscuits	baseline	45.0 (0–780.0)	43.8 (0–330.0)		0.82†
	at 10 weeks	26.7 (0–240.0)	27.2 (0–160.0)		
	change	−18.35	−16.60	1.75	0.91‡

‡Independent Samples T-test, †Mann Whitney U test.

### Changes in primary outcomes

Using independent samples t-tests, significant differences between the intervention and control group in changes in the primary outcomes body weight (−0.35 vs. 0.22 kg, p=0.04), BMI (−0.12 vs. 0.08 kg/m^2^, p=0.04), WC (−0.29 vs. 0.55 cm, p<0.01) and fat mass (−0.99 vs. −0.12%, p<0.01) from baseline to ten weeks were found. From ten weeks to the end of the study period, the decrease in the primary outcomes in the intervention group disappeared, with the exception of “fat mass” (33.76 vs. 34.17%). End point weight (88.28 vs. 87.95 kg), BMI (30.33 vs. 30.22 kg/m^2^) and WC (106.32 vs. 106.16 cm) were again slightly above the baseline values.

Univariate analyses of covariance were performed for changes in the primary outcomes from baseline to ten weeks controlling stepwise for the variables of sex, living situation, smoking habits, alcohol use, SGA drug use, age and duration of stay in sheltered housing. The significant differences between the intervention and control group in changes in weight and BMI disappeared when controlling for duration of stay in sheltered housing (weight: F=2.976, p=0.086; BMI: F=2.820, p=0.094) and SGA drug use (weight: F=3.023, p=0.083; BMI: F=2.997, p=0.085), while it remained significant for changes in WC (stay: F=6.214, p=0.013; SGA: F=6.286, p=0.013) and fat mass (stay: F=6.544, p=0.011; SGA: F=7.076, p=0.008). In the intervention group, a trend was found for a positive relation between duration of stay and weight loss. Participants who had already been staying longer in sheltered housing lost more weight (Spearman’s ρ =−0.11, p=0.08).

In addition, a univariate analysis of covariance was performed for changes in the primary outcomes from baseline to ten weeks, controlling simultaneously for sex, living situation, smoking habits, alcohol use, SGA drug use, and duration of stay in sheltered housing. The significant differences between the intervention and control group in changes in weight (F=3.199, p=0.075) and BMI (F=3.084, p=0.08) disappeared, while it remained for WC (F=6.122, p=0.014) and fat mass (F=8,650, p=0.004) (data not shown).

### Changes in secondary outcomes

In Tables 3 and 4 the changes in the secondary outcomes from baseline to ten weeks are summarized. Differences between the intervention and control group in changes in the secondary outcomes from baseline to ten weeks were only significant for the variable of pedometer determined steps/day (p= <.001). Mean steps per day increased in the intervention group (1256±1933 steps/day), while the number of daily steps in the control group decreased (−426±2754 steps/day). Soft drink intake in the intervention group was decreased at ten weeks (−50.8 g/day), while this was increased in the control group (64.2 g/day). This latter difference between the two groups, however, not statistically significant (p= 0.14). No intervention effect was observed for the other PA variables, food intake, HRQOL and psychiatric symptom severity.

### Adherence to the intervention

A per protocol analysis using analysis of variance was performed to examine the differences in the primary outcomes from baseline to the end of the intervention (at ten weeks) in participants who completed the programme, those who did not, and controls. Completers were defined as individuals in the intervention group who attended at least 8 of 10 sessions (51.2%). In the intervention group, a larger decrease in the primary outcomes was found in those who completed the programme than in those who did not (Table 5). Significant differences were found between completers and controls in changes in body weight (−0.72 vs. 0.22 kg, p=0.03), BMI (−0.23 vs. 0.08 kg/m^2^, p=0.04), WC (−0.64 vs. 0.55 cm, p<0.01), and fat mass (−1.33 vs. 0.05%, p<0.01), but not between non-completers and controls. Participants who completed the programme were significantly less likely to smoke than the non-completers (44.9 vs. 72.7% smokers, p<.001). Compared with those in the intervention group who did not complete the programme, mean age in completers was borderline significantly higher (48.9±11.7 vs. 44.9±12.9 years, p=0.051). All participants in the intervention group who lost at least five per cent of their baseline body weight completed the intervention (10.7%). Thirteen per cent of the completers lost between four and five per cent of their initial body weight (data not shown).

**Table 5:** Baseline assessment and changes in primary outcomes in the intervention group between completers and controls and non-completers and controls from baseline at 10 weeks (per protocol)

**Variable**	**Completers (n=103)**	**Non-completers (n=57)**	**Controls (n=66)**	**∆ Completers vs. Controls**	**p**^**§**^	**∆ Non-completers vs. Controls**	**p**^**§**^
Weight (kg), *mean*±*SD*							
baseline	88.01±19.67	90.39±21.62	83.25±14.78				
at 10 weeks	87.35±19.75	90.34±21.57	83.47±15.38				
change at 10 weeks	−0.72±2.43	−0.05±2.27	0.22±2.40	0.95±0.38	0.03	0.28±0.43	0.80
≥ 5% weight loss, *n (%)*	11 (10.7%)	0	3 (4.5%)				
BMI (kg/m^2^), *mean±SD*							
baseline	30.29±5.67	31.11±6.38	28.87±5.12				
at 10 weeks	30.04±5.74	31.09±6.40	28.95±5.32				
change at 10 weeks	−0.23±0.86	−0.01±0.77	0.08±0.87	0.33±0.13	0.04	0.09±0.15	0.80
Waist (cm), *mean*±*SD*							
baseline	107.54±16.23	107.59±16.67	103.82±13.87				
at 10 weeks	106.87±16.26	107.40±16.47	104.37±14.28				
change at 10 weeks	−0.64±2.41	−0.14±2.42	0.55±2.93	1.23±0.41	<0.01	0.74±0.47	0.25
Fat mass (%), *mean*±*SD*							
baseline	34.82±10.30	33.70±10.09	32.55±11.01				
at 10 weeks	33.41±10.57	33.04±10.09	32.60±11.13				
change at 10 weeks	−1.33±3.24	−0.64±2.44	0.05±2.62	1.45±0.48	<0.01	0.71±0.54	0.39

^§^Univariate Analysis of Variance.

kg, kilogramme; BMI, Body Mass Index; cm, centimetre.

## Discussion

The aim of the study was to examine the effectiveness of a health promotion intervention targeting PA and healthy eating in individuals with MD living in sheltered housing. The study period consisted of a 10-week intervention period followed by a 6-month follow up period.

From baseline to the end of the intervention period (at ten weeks), significant differences in changes in body weight, BMI, WC, and fat mass between the intervention and control group were observed. In the intervention group, a decrease in these outcomes was found, while they increased in the control group. For WC and fat mass, this intervention effect was independent of confounding variables. Within the intervention group, a larger decrease in weight, BMI, WC, and fat mass was found in those who completed the intervention than those who did not. From baseline to the end of the study, the decreases in the intervention group in the primary outcomes disappeared, except for the outcome of fat mass. From baseline to the end of the intervention period, a significant difference between the intervention and control group was observed for the pedometer-determined steps/day. In the intervention group, the mean number of daily steps increased, while it decreased in the control group. No other significant differences between the intervention and control group in changes in the secondary outcomes were found.

The baseline characteristics demonstrated the unhealthy lifestyle behaviour of the study population. For example, a mean BMI of 30 kg/m^2^ was found in our study population, compared with a mean BMI of 25.3 for the general population in Belgium [43]. Smoking prevalence in the study population also greatly exceeded that of the general population in Belgium. Amongst the study population, about 60% were daily smokers, while in the general population it is about 21% [43].

It is well established that individuals with MD are at a greater risk of being overweight or obese than the general population [2,3]. Important reasons for this high prevalence consist of the use of SGAs [9], lower PA levels [10,11] and unhealthy eating habits [15,17]. It is therefore promising that growing attention is being given to the importance of health promotion interventions targeting PA and healthy eating in this population. Several guidelines already emphasized the importance of PA and healthy eating [44-46] and the relevance of health promotion in mental health care is also acknowledged by the European Psychiatric Association [47].

The results of the study demonstrate that relatively small but significant reductions in body weight, BMI, WC, and fat mass are possible following a 10-week health promotion intervention targeting PA and healthy eating. Previous research has shown that weight loss through lifestyle interventions in individuals with MD is possible. The results of these studies must, however, interpreted cautiously due to their methodological limitations such as small sample sizes or the absence of a control group [30,48]. The results of our study are more promising in those participants who completed the intervention. It is also important to note that 68% of the participants in the intervention group were taking a SGA. It is well established that these drugs are associated with weight gain in individuals with MD [8,9].

The decreases in weight, BMI, WC, and fat mass in the intervention group disappeared in the period from the end of the intervention period to the end of the study. So, although emphasis on health promotion targeting PA and healthy eating during a certain period of time is beneficial, it probably needs to be continued. Therefore, the integration of health promotion activities, alongside other treatment aspects such as psychological and medication treatment, into the daily care of individuals with MD should be considered. Lifestyle interventions are essential in lowering the risks and morbidity associated with obesity and should be integrated into the daily care of individuals with MD [49,50].

All mental health nurses involved in the study received the same training by one (the same) member of the research team. They received detailed instructions concerning the delivery of the group-based sessions as well as how to support the participants individually. The aim was to obtain as much consistency as possible concerning the information provided. We are, however, aware that the views and attitudes of individual nurses towards PA and healthy eating may to some extent have influenced the way in which they communicated with the participants.

The design of evaluation studies of public health interventions, like health promotion programmes, poses several problems and they require multiple, flexible, and community-driven strategies [51]. Randomization at the individual level may cause contamination bias if individuals in the control group receive some aspects of the intervention by being in proximity to individuals in the intervention group [52,53]. To avoid the risk of contamination bias arising from the fact that participants in the intervention and control group could be living together in the same SHO, it was decided to use a cluster design with the SHO as the unit of clustering. It was assumed that if SHOs were assigned to a non-preferred study arm, they could be disappointed and their interest in participating in the study could be reduced as a result [23,54]. A substantial risk of non-participation on the SHO-level was also assumed based on the results of previous qualitative research which has identified lack of time due to the high workload in the daily care of patients with MD as a common barrier for mental health nurses to engage in health promotion programmes [55,56]. For these reasons, a preference design appeared to be appropriate. As far as is known to the authors, this is the first trial examining the effectiveness of a health promotion intervention using a cluster preference RCT design.

Besides the significance of the results it is also important to consider their clinical relevance. According to the UK Department of Health [57], reductions in body weight of 5% or more are considered to greatly reduce the risks of physical health problems. At the end of the intervention period (at ten weeks), only 5.5% of the participants in the intervention group reached this target. Among those who completed the intervention, however, the figure jumps to 10.7% with a further 13% losing between four and five per cent of their body weight. To our opinion, integrating PA and healthy eating into the daily care of individuals with MD has the potential to increase the number of them losing at least 5% of their body weight.

The “Health promotion on well-balanced eating and healthy physical activity” program [29] served as the basis for that used in our study. The use of this programme appeared reasonable to us as its target population consists of the general population in the Flanders region (Belgium). Some adjustments were made to it to better meet the needs and interests of the population of individuals with MD included in our study. At this point, it is important to emphasize that, as far as is known to the authors, the efficacy of this general population programme has not been tested, resulting in no information on possible effect size. For this reason, the sample size calculation for our study was performed using a between group change of 3.5 kg found in a systematic review we performed [30]. The mean between group change of 0.57 kg found in our study was yet to a large extent lower as the change of 3.5 kg. To our opinion, two possible explanations for the deviation between the weight loss of 3.5 kg found in our review and the amount of weight loss of 0.57 kg found in our study exist. First, the larger amount of weight loss found in the review may be explained by the fact that all but two of the fourteen studies reviewed consisted of an intervention duration in excess of the 10-week intervention period of our study (range: 2 – 12 months). Second, the studies included in the review consisted of a psycho-educational and/or behavioural intervention. In seven of them, this was combined with supervised exercise. Additionally, in some studies, the intervention also included restricted energy intake/ energy expenditure. This may be a second explanation for the larger amount of weight loss observed in the review. The health promotion programme assessed in our study did not include individualized energy restriction or energy expenditure goals such as low fat or low calorie diets. Based on the results of previous qualitative research [58], it was assumed that such an intervention would be too demanding for both the patients and the mental health nurses. Further research is required to examine the long-term effects of such an intervention (for example: providing the intervention twice a year, intervention with a longer duration).

The sample size calculation, identified that 371 individuals in each group were needed. This sample size was not reached as only 201 individuals in the intervention group and 83 in the control group agreed to participate. This number represents about only 20 per cent of the individuals living in these settings in the Flanders region, which places a limitation on the generalizability of the findings to the wider population of individuals living in sheltered housing. Compared with the most recent available data from the Federal Public Service of Health [59] on the sheltered housing population in the Flanders region, our study population had a higher proportion of women (39 vs. 30%), mean age was slightly lower (46.3±12.3 vs. 50.0±13.0 years), and it was more frequently diagnosed with mood disorders (25 vs. 16%). Data on the duration of stay in sheltered housing (4 vs. 4.2 years) and the proportion of individuals with schizophrenia (38 vs. 39%), substance misuse (16 vs. 18%), and personality disorders (14 vs. 12%) were comparable [59].

The study sample was characterized by high drop out rates. At the end of the study, 40% of the participants in both the intervention and control group were lost to follow-up. The main reason for dropping out was no further interest or motivation to participate. This is congruent with the results of previous research on barriers to individuals with MD engaging in health promotion activities which report lack of motivation and energy as a consequence of the MD and side effects of psychotropic drug use like sedation [60-62].

Another element of concern was the high number of individuals who did not fill out the various questionnaires at the end of the intervention period. This was related to the considerable drop-out rates from the study as a result of the lack of further interest and/or motivation to participate. For example, only 56% in the intervention group and 61% in the control group filled out the SF36 Health Survey questionnaire at the second measurement at ten weeks. Only 46 and 48 per cent of the participants in the intervention and control group respectively registered the number of daily steps during the second registration period (at ten weeks). For this reason, the promising results of the increase in steps/day from baseline to the end of the intervention period must be interpreted cautiously.

We are aware that omitting mixed model analysis is another limitation of the current study. The decision to omit mixed model analysis was based on the fact that a limited group-level variance compared to the total variance was present. For all primary outcomes, an ICC below 5% was found, indicative of a low level of variance at the level of the SHOs [42]. Moreover, the number of participating SHOs and the number of individuals per SHO was below the minimum number of groups and individuals recommended for mixed model analysis [42]. We nevertheless performed unadjusted mixed model analysis to examine whether the SHO clustering had an impact on the intervention effect for the primary outcomes from baseline to the end of the intervention at ten weeks. The significant differences between intervention and control group in changes in body weight (p=0.111) and BMI (p=0.109) disappeared, while they remained significant for WC (p=0.006) and fat mass (p=0.013) (data not shown). No ICC related to health promotion programmes with a cluster randomized controlled design in individuals with MD was found in the literature. To account for the degree of correlation within the several clusters in our study, the calculated sample size was multiplied with a design factor. As for the ICC, no design factor for the type of intervention assessed in our study was found in the literature. So, an assumption was made based on a design effect of 1.5 used in previous studies [32,63].

The target population of our study comprised individuals with a wide variety of psychiatric diagnoses such as schizophrenia, mood disorders, and personality disorders. From a methodological point of view it may have been more suitable to focus only on individuals with a specific diagnosis, which would probably have lead to different results. However, it has already been well established that overweight and obesity affects individuals with MD irrespective of their specific psychiatric diagnosis [1,64,65]. In this sense, health promotion targeting PA and healthy eating appears to be important and desirable for individuals with MD independent of their diagnosis. In any case, sheltered housing is aimed at individuals with a wide variety of mental health problems. For these reasons, it was decided to include individuals irrespective of their diagnosis in the study population. Further research is nevertheless required to examine the effects of this kind of intervention in individuals with a specific psychiatric diagnosis.

## Conclusions

In conclusion, the study described in this paper has shown that small significant improvements in body weight, BMI, WC, and fat mass are possible in individuals living in sheltered housing following a psycho-educational, behavioural and exercise group-based intervention. The results are more promising in those participants who completed the intervention. The results of the present study emphasize the need to integrate lifestyle counselling into the daily care of individuals with MD in order to lower the risk of serious somatic diseases including CVD and type 2 diabetes. Additional controlled trials of longer duration are necessary in order to examine the long-term effects of maintained health promotional efforts. In this context, research on the optimal “intervention dose” in terms of acceptability, effectiveness and cost-effectiveness of the intervention is also required. Further research is additionally required to examine the effectiveness of such programmes in other settings, for example inpatient settings, and under other conditions such as group-based versus individually-based interventions or programmes limited to individuals with BMI>25 kg/m^2^ or on the same medication regimen. Finally, in view of the high drop-out rates in our study population, further research is also needed to examine the most effective ways to motivate individuals with MD to participate and persist in health promotion programmes.

## Competing interests

The authors declare that they have no competing interests.

## Authors’ contributions

NV and LA coordinated the study. NV, LM, LA, DDB were responsible for the conception and design of the study. NV collected the data. NV, EC, DDB, and CV did the data analysis. NV, EC, CV, JDM, LM, CVH, DDB, and LA contributed to interpretation of the findings and critical revision of the manuscript. All authors read and approved the final manuscript.

## Pre-publication history

The pre-publication history for this paper can be accessed here:

http://www.biomedcentral.com/1471-2458/13/657/prepub
